# The First Successful Endovascular Management of Posterior Fossa Arteriovenous Malformations in Nepal: Case Series

**DOI:** 10.31729/jnma.4933

**Published:** 2020-12-31

**Authors:** Subash Phuyal, Pooja Agrawal, Kapil Dawadi, Raju Paudel, Ritesh Lamsal

**Affiliations:** 1 Department of Neuroimaging and Intervention Neuroradiology, Grande International Hospital, Dhapasi, Kathmandu, Nepal; 2 Department of Radiology, Norvic International Hospital, Thapathali, Kathmandu, Nepal; 3 Department of Neurology, Grande International Hospital, Dhapasi, Kathmandu, Nepal; 4 Department of Anaesthesiology, Institute of Medicine, Tribhuvan University, Maharajgunj, Kathmandu, Nepal

**Keywords:** *arteriovenous malformation*, *embolization*, *therapeutic*, *intervention*, *neurology*

## Abstract

Posterior fossa arteriovenous malformations represent 7-15% of all intracranial AVMs. They carry a higher risk of rupture than supratentorial AVMs and are associated with considerable rates of morbidity and mortality. Available treatment options include conservative management, microsurgical resection, radiosurgery, endovascular embolization, or combinations of these modalities. Recent advances in endovascular techniques have revolutionized their management with better clinical outcomes. We illustrate two cases of posterior fossa AVMs treated by endovascular techniques with good clinical outcomes. The first patient also had associated flow-related aneurysms. One of these aneurysms had already ruptured, so it was coiled first followed by AVM nidus embolization using the same microcatheter. The second patient had a diffuse type of posterior fossa AVM for which staged-embolization was planned and the first-stage partial embolization was successfully performed.

## INTRODUCTION

Posterior fossa arteriovenous malformations constitute only 7 to 15% of all intracranial AVMs.^1^ Conventional surgical treatment of posterior fossa AVM is associated with significant mortality (6%) and morbidity (16%) due to the complex surgical anatomy and its proximity to important neural and vascular structures.^2^ However, recent advances in endovascular techniques have transformed conventional management and improved outcome. Endovascular embolization is an effective treatment option, although multiple sessions are sometimes necessary to achieve complete obliteration. Here, we report two such cases of posterior fossa AVMs that were successfully treated by endovascular techniques for the first time in Nepal with good post-procedure outcomes.

## CASE REPORT

### CASE 1

**Figure 1:**
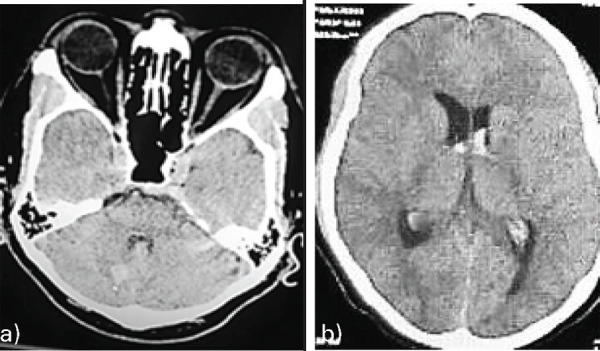
Axial plain NCCT images (a and b) of brain soft tissue window showing focal right cerebellar bleed with the extension of the bleed into the ventricular systems without hydrocephalus.

A 39-year-old male patient presented in the emergency department with sudden-onset severe headache for two days. On examination, no neurological deficit was noted. A plain non-contrast computed tomography (NCCT) revealed right cerebellar bleed with intraventricular extension (Figure 1).

**Figure 2:**
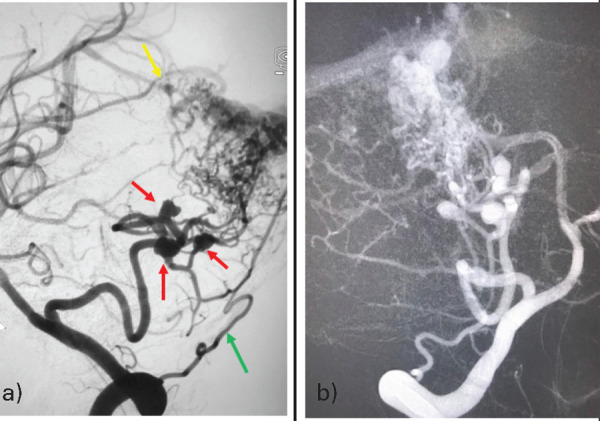
2-D right vertebral artery imaging shows predominant feeding from the hypertrophied right PICA which has three associated flow-related saccular aneurysms (red arrows). The AVM nidus also had a supply from the right superior cerebellar artery (yellow arrow) and the right posterior meningeal artery (green arrow). There was single venous drainage into the right transverse sinus.

A CT-angiography showed posterior fossa AVM in the right cerebellar hemisphere with feeders from the right posterior inferior cerebellar artery (PICA) and three flow-related saccular aneurysms. On the second day of admission, the patient was taken up for endovascular embolization under general anesthesia. A 6-F right femoral access was taken and a diagnostic cerebral angiogram (DSA) was performed through a 5-F Picard catheter. The DSA confirmed the presence of predominant arterial flow from the hypertrophied right PICA, which had three flow-related saccular aneurysms. The AVM nidus also had some supply from the right superior cerebellar artery and the right posterior meningeal artery. There was single venous drainage into the right transverse sinus with no venous stenosis or ectasia (Figure 2). After defining the arterial feeders, the draining vein, and the aneurysms along with the feeders, we planned to coil the feeding artery aneurysms, and subsequently embolize the nidus.

**Figure 3:**
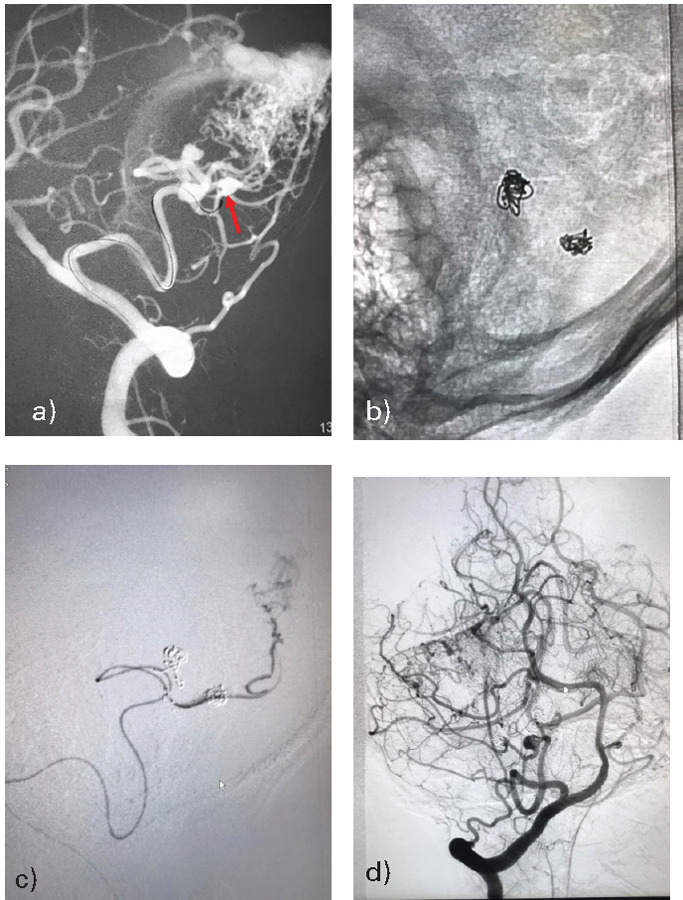
a) A 2-D left vertebral artery angiogram showing microcatheter tip in the aneurysm sac (red arrow) b) detachable coils in two flow-related saccular aneurysms c) The same microcatheter was navigated distally into the AVM nidus d) Final angiographic run showing near-complete obliteration of AVM nidus.

A 6-F Envoy guiding catheter (Codman) was placed in the distal part of the V-2 segment of the right vertebral artery. Subsequently, Headway Duo microcatheter (Microvention) was navigated over traxcess (Microvention) micro-guidewire, and two feeding artery aneurysms with unfavorable morphology (irregular outline) were embolized using detachable coils (Figure 3). The same Headway Duo microcatheter (both coil- and onyx-compatible) was navigated distally to reach proximate the nidus. After confirming the position, the AVM was embolized using onyx-18. A control angiogram revealed a near-complete reduction in the size of the AVM nidus (Figure 3). The entire procedure was uneventful. The patient was extubated immediately after the procedure and shifted to the intensive care unit (ICU) for observation. His post-operative recovery was uneventful and a repeat CT scan did not show any hydrocephalus. The patient was discharged from the hospital on the seventh day of admission without any neurological deficit.

### CASE 2

**Figure 4:**
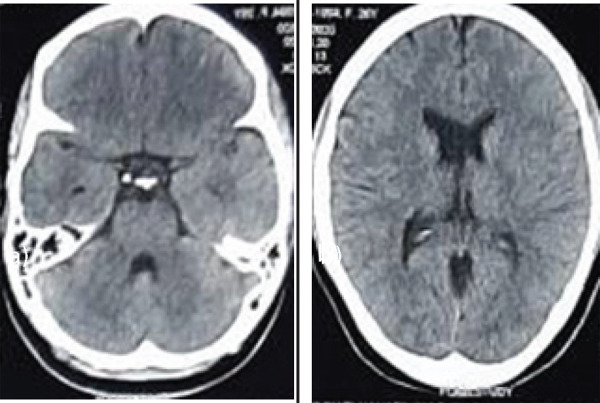
Axial plain NCCT images (a and b) of the brain soft tissue window shows no parenchymal or intraventricular bleed.

A 26-year-old female patient presented with headache and vertigo for six months. On examination, there was no neurological deficit. A plain NCCT was normal (Figure 4). CT-angiography showed posterior fossa AVM in the left cerebellar hemisphere with feeders from the left superior cerebellar artery (SCA) and the left PICA. The venous drainage was in the left transverse-sigmoid junction.

**Figure 5:**
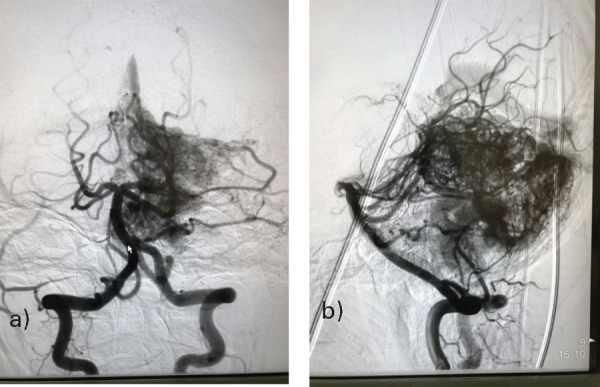
(a and b) 2-D right and left simultaneous vertebral arteries angiographic runs shows diffuse left cerebellar AVM with feeders from left SCA and left PICA. There was single venous drainage into the left transverse-sigmoid sinus junction.

After written informed consent, the patient was up taken for endovascular embolization under general anesthesia. A 6-F femoral access was taken and DSA was done through a 5-F Picard catheter. The DSA confirmed the presence of left cerebellar fossa AVM with feeders from the left SCA and left PICA. There was single venous drainage into the left transverse-sigmoid sinus junction with no venous stenosis or ectasia (Figure 5).

**Figure 6:**
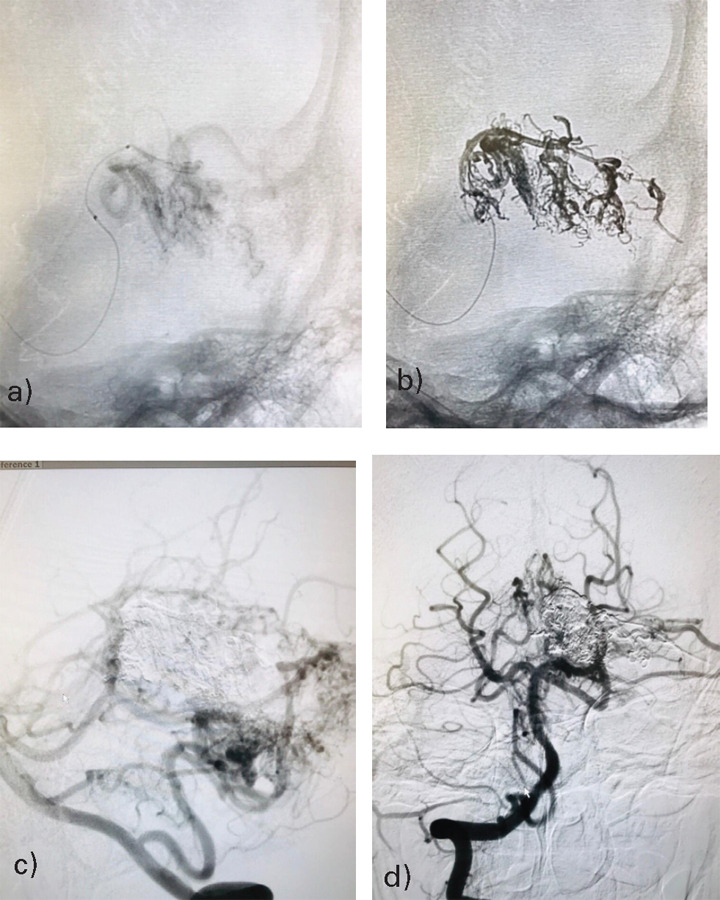
(a) Microcatheter run from the left distal SCA feeder placing catheter near the AVM nidus (b) Onyx embolization from the microcatheter (c and d) Final angiographic run showing 50-60% obliteration of the AVM nidus.

We decided to perform partial embolization of the AVM. A 6-F Envoy guiding catheter (Codman) was placed in the distal part of the V-2 segment of the left vertebral artery. Subsequently, Apollo onyx delivery microcatheter (Medtronic) was navigated over mirage 0.08" (eV3) micro guidewire into the left SCA feeder distally to reach the nidus. After confirming the position, the AVM was embolized using onyx-18 (eV3). Control angiogram revealed an approximately 50-60% reduction in the size of AVM nidus (Figure 6). The entire procedure was uneventful. His post-operative recovery was uneventful and repeat CT-scans were normal. The patient was discharged from the hospital on the sixth day of admission. She was advised for a follow-up angiogram and second-stage embolization of PICA-feeder after 3 months.

## DISCUSSION

Posterior fossa AVMs are complex neurovascular lesions that have several therapeutic challenges. The first posterior fossa AVM surgery was done in 1932 and since then many small case series describing its management have been published.^3^ Current treatment options include conservative management, microsurgical resection, radiosurgery, endovascular embolization, or combinations of these modalities.^4^

Posterior fossa AVM has a higher risk of hemorrhagic presentation compared with supratentorial AVM.^5^ Hemorrhage is the presenting symptom in nearly half of the patients with posterior fossa AVMs. This is followed, in decreasing order of frequency, by seizure (33%-46%), headache (14%-34%), or progressive neurological deficit (21%-23%).^3^ Other risk factors for AVM rupture are previous hemorrhage, deep location, anatomic variations of the lesion, such as high feeding artery pressure, feeding artery aneurysms, deep venous drainage, and small lesion size.^5^ CT, magnetic resolution imaging scans, and DSA of the cerebral vessels are necessary to plan the treatment. Cerebral DSA is extremely important as it precisely defines the number of feeding arteries, their relationship to the cortical branches supplying the surrounding normal brain, the exact size of the nidus, the presence of associated aneurysms, and the type and caliber of venous drainage.

AVMs are classified using the Spetzler-Martin classification scheme, which is based on three radiological characteristics: size, location, and the pattern of venous drainage.^6^ However, for posterior fossa AVMs, several other methods of classifications are also described. Based on the location, the infratentorial AVMs can be classified into eight groups as midbrain, pontine, medullary, sub-occipital, petrosal, tentorial, vermian, and tonsillar. Again, brainstem AVM can be further classified according to their location as mesencephalic (supplied by the SCA), pontine (supplied by the anterior-inferior cerebellar artery, or medullary (supplied by the PICA).^7^ Brainstem AVMs can also be classified according to the depth of the lesion as either pial (superficial) or parenchymal.^7^ The choice of treatment depends on the characteristics of each case. AVMs of Spetzler-Martin grades I-III can be successfully treated with neurosurgical excision with a low risk of morbidity (0%-4.2%).^8^ On the other hand, Spetzler-Martin grade IV and V lesions have a high surgical risk and endovascular therapy is preferred in these lesions, either alone, or in combination with microsurgery or stereotactic radiosurgery.^9^ AVMs located in the periphery of the cerebellar hemispheres, lower vermis, tonsils, and the pial surface of the brainstem can be treated neurosurgically with low rates of morbidity.^10^ In contrast, patients with lesions at the deep cerebellar nuclei or brainstem parenchyma are not suitable for surgery because of the proximity to important centers, such as cranial nerve nuclei and major motor pathways, with the potential to disrupt the perforating branches of the vertebrobasilar system.

Endovascular embolization can also be used as an adjuvant treatment to reduce the volume of the nidus before microsurgery or stereotactic radiosurgery. The overall goal of AVM therapy is the complete removal or obliteration of the AVM, although partial therapy may confer palliative benefits for headache, seizure, and progressive neurological deficits. Complications of endovascular therapy include embolic stroke, intracerebral hemorrhage, pulmonary embolism, and microcatheter retention. The reported complication rates vary from 3% to 25%, but serious neurological events or death occur in only 3%-8% of cases. Staged embolization of large AVMs, meticulous technique, and rapid withdrawal of the microcatheter after injection greatly reduce the risk of complications.

To the best of our knowledge, these two cases are the first described cases of posterior fossa AVMs, which were successfully treated by endovascular techniques in Nepal with good post-procedure outcomes. Considering gradual advancement in embolization techniques and better post-treatment outcomes, embolization techniques will likely be preferred to surgery in many similar cases in Nepal in the days ahead.
